# Editorial: The increasing relevance of traditional medicine systems for the primary health care sector and general practice: global research perspectives, volume II

**DOI:** 10.3389/fmed.2026.1820234

**Published:** 2026-04-17

**Authors:** Rammanohar Puthiyedath, Anand Dhruva, Pathirage Kamal Perera, Christian S. Kessler

**Affiliations:** 1 Amrita School of Ayurveda, Amrita Vishwa Vidyapeetham, Amritapuri, Kerala, India; 2 Division of Hematology and Oncology, Department of Medicine, University of California, San Francisco, San Francisco, CA, United States; 3 Osher Center for Integrative Health, University of California, San Francisco, San Francisco, CA, United States; 4 Faculty of Indigenous Medicine, University of Colombo, Colombo, Sri Lanka; 5 Department of Integrative Health Care and Prevention, University of Augsburg & University Hospital Augsburg, Augsburg, Germany; 6 Department of Internal Medicine and Nature-Based Therapies, Immanuel Hospital Berlin, Berlin, Germany

**Keywords:** health policy, integrative medicine, primary care, public health, traditional medicine, universal health coverage

Traditional, Complementary and Integrative Medicine (TCIM) is becoming a vital component of health systems in the global health discourse. Chronic lifestyle disorders, aging populations with increasing disease burden, persisting and emerging infectious diseases and sequelae are overwhelming primary health care systems globally (1). With its pluralistic outlook and person-centered approach, TCIM offers concepts embedded in various cultures that can complement biomedical care. While already in use by a significant number of stakeholders worldwide, traditional medicine is now garnering the attention of policy makers, regulators, clinicians and researchers for health promotion, prevention, chronic care and rehabilitation (2).

The recent Second Global Summit on Traditional Medicine of the World Health Organization that was conducted in New Delhi in December 2025 (3) highlights the importance of this topic. The Delhi Declaration (4) that marked the culmination of this event reiterated global commitments to integration of traditional medicine into health care systems on the foundations of evidence-informed, safe and effective approaches. It accentuated the importance of research capacity building, alignment of regulatory policies with epistemologies of traditional medicine, highlighting the scope of traditional medicine to contribute to universal health coverage and sustainable development goals. The Delhi Declaration carries forward the momentum of the earlier 2023 Gujarat Declaration and this policy landscape provides a perfect backdrop for the present Research Topic of articles on traditional medicine.

The current volume brings together 33 peer reviewed articles that cover Ayurvedic Medicine, Chinese Medicine, Korean Medicine, Kampo Medicine, Acupuncture, European TCIM, and Mind-Body practices especially emphasizing public health oriented and integrative lifestyle interventions. Striking is the methodological diversity including clinical trials, real world and registry data, study protocols, bibliometric analyses, and systematic reviews. Notable is the attempt to adhere to the epistemological foundations of traditional medicine while engaging with and in the framework of contemporary evidence-based approaches in health care and beyond. The Research Topic underpins the wide scope of TCIM and its emerging role especially in primary health care and patient-centered models across disparate socio-cultural settings. Below is a summary of the papers included in this volume.

## Ayurvedic medicine

1. Learning technologies for capacity building in traditional medicine (Nedungadi et al.)

This educational research introduces and evaluates digital learning technologies designed to enhance clinical reasoning and research competencies in traditional medicine education. User feedback indicates high acceptability and perceived educational value. The study addresses scalability and training challenges in contemporary Ayurveda education.

2. Integrative Ayurveda for chemotherapy-induced hand–foot syndrome (Sharma et al.)

This exploratory study assesses an Ayurvedic add-on protocol for managing chemotherapy-induced hand–foot syndrome. Patients receiving integrative care reported reduced pain and improved quality of life without disruption of oncological treatment. The findings support Ayurveda's role in supportive integrative oncology.

3. Sirāvyadha and inflammatory biomarkers in varicose veins (Sivadas et al.)

This exploratory clinical study investigates the effects of Sirāvyadha (venesection) on inflammatory markers in varicose vein patients. Reductions in interleukin-6 levels were accompanied by symptomatic improvement. The study links classical Ayurvedic procedures with measurable biological outcomes.

4. Evaluating Prakriti Assessment Tools in Ayurveda (Venkatesh et al.) This review analyzes 64 Prakriti Assessment Tools (1987–2024) to assess their scientific validity. Among 94 studies, only 20 tools showed any validation, and just two met most methodological standards. While efforts toward objectivity and technology integration are evident, major gaps remain in reliability, dimensionality testing, and population diversity. Although several biological correlates of Prakriti have been explored, they are not well integrated into tools. Overall, Prakriti assessment remains underdeveloped and requires more rigorous validation to support its use in personalized healthcare.

5. Ayurvedic preconditioning before IVF (protocol) (Muraleedharan et al.)

This study protocol outlines an exploratory trial evaluating Ayurvedic treatment as a preparatory intervention before IVF in women with diminished ovarian reserve. Clinical outcomes are combined with hormonal assessments and multi-omics analysis of follicular fluid. The protocol represents a transdisciplinary approach to integrative reproductive medicine.

## Traditional Chinese medicine (TCM)

6. Acupuncture for post-operative gastroparesis syndrome (Xv et al.)

This systematic review and meta-analysis synthesizes randomized controlled trials evaluating acupuncture as an adjunctive therapy for post-operative gastroparesis syndrome. The findings indicate improvements in gastric emptying, overall clinical response rates, and post-operative recovery parameters compared to conventional management. However, variability in study quality, reporting standards, and safety documentation underscores the need for more rigorously designed trials.

7. Auricular acupoint therapy for functional gastrointestinal disorders (Shen et al.)

This meta-analysis examines the effectiveness of auricular acupoint therapy in patients with functional gastrointestinal disorders. The pooled evidence suggests significant reductions in symptom severity, alongside improvements in anxiety and depressive symptoms commonly associated with these conditions. The study highlights auricular therapy as a low-cost, non-invasive intervention with potential relevance for primary care settings.

8. Acupuncture for musculoskeletal pain: an evidence map (Ang et al.)

This comprehensive evidence map collates findings from systematic reviews and meta-analyses addressing acupuncture for a wide range of musculoskeletal pain conditions. While consistent short-term pain relief is reported across many indications, the analysis reveals substantial heterogeneity and generally low methodological quality in the existing review literature. The work provides a strategic overview of evidence gaps and priorities for future research.

9. Peripheral–central correlation study of acupuncture for chronic tinnitus (protocol) (Jiang et al.)

This study protocol outlines a randomized controlled trial designed to investigate the mechanisms of acupuncture in chronic tinnitus. By integrating peripheral auditory assessments with central neurophysiological measures and patient-reported outcomes, the study aims to bridge clinical efficacy with mechanistic understanding. The protocol reflects increasing methodological sophistication in acupuncture research.

10. Tongue manifestations for diagnosing primary Sjögren's syndrome (Chen et al.)

This original research develops and validates a diagnostic model based on tongue manifestations derived from traditional Chinese medicine. The model demonstrates high accuracy and discrimination for identifying primary Sjögren's syndrome, offering a non-invasive screening approach. The study illustrates how classical diagnostic concepts can be operationalized using modern statistical and clinical validation methods.

11. Chinese herbal medicine for chronic pain: a bibliometric analysis (Wang et al.)

This bibliometric analysis maps global research trends on Chinese herbal medicine for chronic pain management over more than a decade. The findings show a steady increase in publications, expanding international collaboration, and growing use of network pharmacology and molecular docking to explore mechanisms of action. The study situates herbal medicine research within broader translational and systems-biology frameworks.

12. Chuzhen therapy for lumbar disc herniation (case report) (Hu et al.)

This case report describes the long-term clinical and radiological course of a patient with lumbar disc herniation treated using Chuzhen therapy. Serial imaging demonstrated substantial regression of disc herniation alongside sustained symptom improvement. While limited by its single-case design, the report highlights conservative traditional manual therapy as a potential non-surgical option.

13. Factors influencing the use of TCM in cancer care (Xu et al.)

This qualitative meta-synthesis explores patient and provider perspectives on the integration of traditional Chinese medicine into cancer care. The analysis identifies key facilitators, such as symptom management and patient demand, as well as barriers including communication gaps, limited practitioner knowledge, and institutional constraints. The study shifts attention from efficacy to real-world implementation challenges.

14. Eight Constitution Acupuncture and diet for functional dyspepsia (Cho et al.)

This case series presents individualized management of functional dyspepsia using Eight Constitution Acupuncture combined with constitution-specific dietary recommendations. Patients demonstrated symptomatic improvement and enhanced quality of life. The study illustrates personalized, constitution-based approaches central to traditional East Asian medicine.

15. Acupuncture and mortality in heart failure among people with disabilities (Jun et al.)

Using nationwide health insurance data, this cohort study investigates the association between acupuncture use and mortality in patients with heart failure and disabilities. Acupuncture exposure was associated with lower all-cause mortality after adjustment for confounders. The study highlights the potential role of acupuncture as an adjunct in chronic disease management at the population level.

16. Electroacupuncture for Vertebral Fracture Recovery, (Yeum et al.)

This case report shows that electroacupuncture applied to deep paraspinal muscles improved pain, mobility, and quality of life in two patients with vertebral compression fractures, suggesting a minimally invasive approach that may enhance bone healing and functional recovery.

## Korean medicine

17. Integrative Korean Medicine for traffic accident–related neck and low back pain (Kim et al.)

This retrospective inpatient study examines outcomes of integrative Korean Medicine treatment for musculoskeletal pain following traffic accidents. Significant improvements were observed not only in pain and functional disability but also in associated psychological symptoms such as anxiety and sleep disturbance. The findings support a biopsychosocial approach to pain management.

18. Utilization of Korean Medicine inpatient care (Lee et al.)

This health panel analysis explores patterns and determinants of inpatient Korean Medicine utilization. Musculoskeletal conditions emerged as the dominant indication, and patient satisfaction remained high despite limited insurance coverage. The study provides policy-relevant insights into traditional medicine use within an integrated national health system.

## Japanese medicine (Kampo)

19. Kampo medicine for long COVID (Ono et al.)

This prospective observational study assesses the impact of Kampo medicine on patients experiencing long COVID symptoms. Improvements were observed in health-related quality of life, particularly in cognitive and fatigue-related domains. The findings contribute early real-world evidence for traditional medicine approaches in post-viral syndromes.

## Traditional European medicine

20. Hydrotherapy and acupressure for restless legs syndrome (RCT) (Kubasch et al.)

This randomized pilot trial evaluates self-applied Kneipp hydrotherapy and acupressure in adults with restless legs syndrome. Both interventions were feasible, safe, and associated with trends toward improved symptom severity and disease-specific quality of life. The study supports low-cost, self-care–oriented interventions in chronic neurological conditions.

21. Hydrotherapy and acupressure for restless legs syndrome (qualitative study) (Siewert et al.)

As a qualitative complement to a clinical trial, this study explores patient experiences with hydrotherapy and acupressure. Participants reported varying benefits, ease of integration into daily routines, and practical challenges. The findings provide contextual insights into real-world applicability.

22. Reflexology for preoperative anxiety (Samuel et al.)

This randomized controlled trial investigates reflexology as an adjunct for reducing preoperative anxiety. Modest but significant reductions in anxiety were observed compared to standard care. The study supports reflexology as a low-risk supportive intervention in perioperative settings.

23. Kneipp Health Concept in German kindergartens (Ortiz et al.)

This cluster-randomized mixed-methods study evaluates the Kneipp Health Concept in early childhood education settings. The intervention integrates hydrotherapy, physical activity, nutrition, herbal care, and lifestyle education. The study extends traditional European medicine into preventive and community health promotion.

## Mind–body and movement-based traditions

24. Meditation for cardiovascular outcomes in high-risk populations (Norris et al.)

This randomized trial with long-term follow-up examines meditation for cardiovascular risk reduction. While short-term surrogate markers showed limited change, long-term follow-up revealed reductions in major cardiovascular events. The study highlights the importance of extended outcome horizons in mind–body research.

25. Global trends in Tai Chi research (2004–2024) (Li et al.)

This bibliometric analysis maps two decades of Tai Chi research across health and rehabilitation domains. The findings demonstrate sustained growth, expanding interdisciplinary collaboration, and increasing relevance to aging, balance, and chronic disease prevention.

## Integrative, policy, and cross-system studies

26. AYUSH integration into India's primary health care (Nesari et al.)

This policy review documents India's large-scale integration of AYUSH systems into primary health care infrastructure. The analysis highlights institutional frameworks, workforce deployment, and alignment with national health programs. The paper presents a functioning national model of integrative care.

27. Integrative medicine for hospital staff during war (Schiff et al.)

This qualitative study examines integrative medicine interventions provided to hospital staff during wartime. Participants reported improved emotional resilience, functional capacity, and professional meaning. The findings extend TCIM's relevance to occupational health and crisis contexts.

28. Experiences with complementary medicine in mental health care (Siewert, Wackermann et al.)

This qualitative substudy explores patient experiences with complementary and integrative medicine in psychiatric and psychosomatic care. Responses ranged from symptom relief to ambivalence, underscoring the importance of context, readiness, and therapeutic guidance. The study foregrounds patient-centered perspectives.

29. Drug–herb interactions in primary care (Sujithra et al.)

This focused review addresses the clinical challenge of drug–herb interactions in primary care practice. It synthesizes evidence on common risks and proposes pragmatic screening and management strategies. The article reinforces the importance of safety and pharmacovigilance in integrative care.

30. *Rosa damascena* extract for sexual function in breastfeeding women (Akbarzadeh et al.)

This randomized controlled trial evaluates the effects of *Rosa damascena* extract on sexual function and anxiety in breastfeeding women. Significant improvements were observed across multiple domains of sexual function, along with reductions in trait anxiety. The study supports traditional herbal approaches in postpartum care.

31. CBT with adjunct Brahmi for premenstrual syndrome (protocol) (Khan et al.)

This protocol describes a randomized trial combining cognitive behavioral therapy with Brahmi supplementation for premenstrual syndrome. The study integrates psychological and herbal approaches using validated outcome measures. It exemplifies rigorously designed integrative mental health research.

32. TCIM-Based Workplace Health Needs in Nursing Care (Voigt et al.)

A participatory qualitative assessment in a long-term care facility identified high physical and psychological stress among nursing staff alongside existing coping strategies, including TCIM practices. The study shows that involving staff in needs assessment is key to designing effective, sustainable, and context-specific workplace health promotion programs.

33. Indigenous Knowledge and TCIM for Planetary Health, (Seifert et al.)

This review highlights the role of Indigenous Knowledges for Health (IKH) and Traditional, Complementary, and Integrative Medicine (TCIM) in advancing holistic well-being and planetary health. It argues that modern health systems are limited by reductionist approaches and calls for integration of traditional knowledge systems that emphasize interconnectedness between humans, society, and the environment. The paper outlines how IKH and TCIM can inform sustainable healthcare, policy frameworks, and innovation in areas such as personalized medicine and prevention. Overall, it advocates for a pluralistic, integrative model to achieve health equity, sustainability, and global well-being.

## Uniqueness of this volume

As a Research Topic, these 33 papers clearly indicate a shift from reductionistic efficacy focused studies to more system aware, context sensitive and implementation oriented approaches exploring the contemporary relevance of TCIM. Noteworthy is the emphasis on real-world clinical practice and integration with primary health care realities even as attempts are made to strengthen relevance and rigor of methodology and transparency.

The papers included in this Research Topic demonstrate that TCIM is increasingly being harnessed to address gaps in (chronic) disease management in areas like integrative oncology, mental health, preventive health and fields like workforce wellbeing. Some of the papers engage with education, digital tools, policy frameworks and professional capacity building. This is a welcome balance between implementation and therapeutic application. From this viewpoint, this volume is well-aligned with the current global priorities in health care (WHO-frame) that positions TCIM as anindispensable component to nurture inclusive and person centered primary health care frameworks (5).

While this Research Topic can foster the much needed dialogue amongst stakeholders on how traditional medicine can be meaningfully integrated into global health care systems, it already provides a panoramic overview of the evolving mindscape of TCIM that can offer the much needed support for policymakers, educators, researchers and clinicians to engage with it meaningfully.

In these articles, we see aneffort to transcend the binary of “modern vs. traditional” by embracing an approach that seeks to deploy methodological bridges. Especially, it is important to note that models focused on single-molecule pharmacology are most often inappropriate for assessment of traditional medicine. The diversity of traditional medical systems and practices represented in this volume are complex in approach and systems based, operating on the basis of pattern differentiation in clinical assessment that results in individualized prescriptions and multimodal interventions with continuous evaluation over time and dynamic modification of the treatments. Many papers in this Research Topic indicate such a shift with a focus on pragmatic trials, mixed methods designs, registry-based cohort studies, and implementation research. Such research designs are better suited to capture real world clinical practice characterized by the intersection of multi-morbidity, polypharmacy, and lifestyle factors. It is encouraging to see that investigators are shifting attention from isolating components of whole and complex interventions and are increasingly focusing on the lived experience outcomes in terms of efficacy/effectiveness, safety, acceptability, and health economics in the given contexts.

Of those, a major concern in the context of integration of traditional medicine at primary health care level is safety. Several oncology related articles in this volume address the challenges and outcomes of concomitant use of traditional medical interventions along with standard of care and the review on drug-herb interactions stresses the importance of establishing a robust pharmacovigilance system in primary health care settings.

While alternatives to reductionistic research designs are critical, it is equally important to ensure rigor in research studies on traditional medicine. Major concerns are heterogenous reporting standards, variable quality of herbal formulations, inadequate reporting of adverse events, lack of blinding and randomization where it is feasible. In order to address these concerns, extensions of reporting guidelines like CONSORT is called for. On the other hand, some of the papers in this volume integrate omics, network pharmacology and systems biology. This represents the much needed attempts to link theoretical constructs in traditional medicine with measurable biological correlates.

Workforce competencies are critical to enable TCIM to contribute to universal health coverage by quality-assured TCIM care. The paper on learning technologies for traditional medicine promises to fill this gap by upscaling training of healthcare professionals. Indeed, educational innovation is critical in primary health care settings and there are fertile areas like development of clinical decision aids and data capturing tools based on diagnostic categories and standardized terminologies grounded in TCIM.

It is encouraging to see the public health orientation of many contributions to this volume. Traditional foods systems and school-based health promotion initiatives are examples illustrating the scope of prevention with TCIM. Preventive frameworks of TCIM like daily routine, seasonal adaptation, balanced diet and stress regulation align well with the contemporary emphasis on social determinants of health and behavioral risk factors.

As we review the contributions to this volume, it becomes apparent that many research needs and priorities remain to be addressed. Long term outcome data and comparative effectiveness studies are essential to understand the benefits of integrative approaches over standard of care, especially in real-world settings and in health economics contexts. There is a substantial need to allow and ensure methodological rigor and epistemic diversity at the same time. The apparent contradiction can be resolved through the specific further development of scientific methodological concepts and tools for the TCIM field. This second volume invites to envision a pluralistic global health system that is firmly grounded in science, sensitive to cultural diversity and is focussed not on disease management alone, but also on long term health.
